# Killing of Microbes and Cancer by the Immune System with Three Mammalian Pore-Forming Killer Proteins

**DOI:** 10.3389/fimmu.2016.00464

**Published:** 2016-11-03

**Authors:** Eckhard R. Podack, George P. Munson

**Affiliations:** ^1^Department of Microbiology and Immunology, Miller School of Medicine, University of Miami, Miami, FL, USA

**Keywords:** complement, C9, Perforin-1, Perforin-2, MPEG1, cytotoxic T cells, natural killer cells, pore-forming protein

## Abstract

Immunology is the science of biological warfare between the defenses of our immune systems and offensive pathogenic microbes and cancers. Over the course of his scientific career, Eckhard R. Podack made several seminal discoveries that elucidated key aspects of this warfare at a molecular level. When Eckhard joined the complement laboratory of Müller-Eberhard in 1974, he was fascinated by two questions: (1) what is the molecular mechanism by which complement kills invasive bacteria? and (2) which one of the complement components is the killer molecule? Eckhard’s quest to answer these questions would lead to the discovery C9 and later, two additional pore-forming killer molecules of the immune system. Here is a brief account of how he discovered poly-C9, the pore-forming protein of complement in blood and interstitial fluids: Perforin-1, expressed by natural killer cells and cytotoxic T lymphocytes; and Perforin-2 (MPEG1), expressed by all cell types examined to date. All the three killing systems are crucial for our survival and health.

## Introduction

The immune system is faced with the difficult tasks of surveillance and elimination of pathogenic microbes and cancers. The elimination of the invaders – here defined broadly as cancers, viruses, extracellular, or intracellular bacteria – can be achieved by a *physical process* involving the insertion of clusters of pores on targeted membranes that perforate the bacterial envelope or damage the cytosolic membranes of infected or cancerous cells. These assaults disrupt the permeability barrier that is required for maintaining the life of all cells. By itself, barrier disruption by pore formation can be lethal if it is sufficiently extensive. Less extensive pore formation may nonetheless lead to the destruction of the targeted entity by facilitating the delivery of ancillary lethal agents to sensitive sites of the targeted cell or bacterium.

The evolution of three pore-forming proteins was probably driven by the need to assure destruction of invaders, regardless of the type of pathogen, or location of the invasion. Different – but sometimes overlapping and perhaps complementary – strategies are required by the immune system to eliminate extracellular bacteria (complement and poly-C9), intracellular viruses and cancer cells (Perforin-1), and intra- and extracellular bacteria (Perforin-2). Through his interest in biological killing, Eckhard R. Podack discovered all three pore-forming proteins of the mammalian immune system and defined their critical importance in immune defense. Here is a brief account of his hypotheses that resulted in the discovery of these critical immune effectors and their function in the context of other important developments in science.

### The Molecular Mechanism of Pore Formation

The analogy to human warfare of immune destruction of pathogenic invaders by pore-forming proteins is obvious. The most effective way of killing an enemy is by the *bullet of a gun*, creating a potentially lethal hole by physical forces. Machine gunning is even more effective by creating multiple holes in the target. By analogy, the three pore-forming proteins of the immune system, such as C9, Perforin-1, and Perforin-2, are the bullets creating direct and immediate physical damage by inserting clusters of pores or holes (machine gunning) of large inner diameter (10–16 nm) into the envelope of the targeted microbes or cells, respectively. The destructive bullets of the immune system, the pore-forming proteins, must be carefully targeted by use of specialized “guns” depending on the nature and location of the invader. The bullet of the immune system, such as the bullet of a gun, is blind as to its target and kills whatever it hits. Collateral damage has to be minimized.

**Figure 1:**
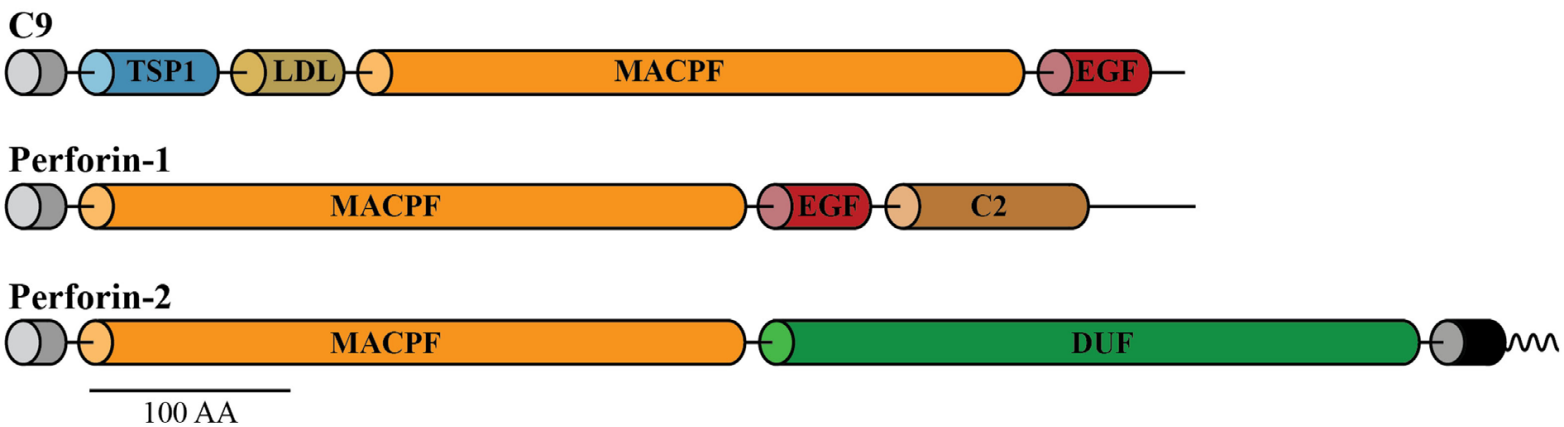
**Domain organization of C9, Perforin-1, and Perforin-2**. All the three pore-forming proteins contain amino-terminal signal peptides (gray cylinders) and membrane attack complex perforin (MACPF) domains. Unlike the soluble proteins C9 and Perforin-1, Perforin-2 is a type I membrane protein with a membrane spanning alpha helix (black cylinder) near its carboxy terminus. The short cytosolic tail (wavy line) of Perforin-2 is ubiquitylated by a CRL in response to PAMPs such as LPS. Perforin-2 also contains a domain of unknown function (DUF) that is conserved among Perforin-2 orthologs. Domain architecture was retrieved from UniProt entries P02748, P14222, and Q2M385. Abbreviations: TSP1, thrombospondin type-1 repeat; LDL, low-density lipoprotein receptor class A repeat; EGF, epidermal growth factor-like domain; C2, calcium-dependent phospholipid-binding domain. This figure was adapted from McCormack et al. (1).

As mentioned earlier, perforation of the cell membranes or bacterial envelopes is achieved by the formation of dense clusters of large pores of 10–16 nm inner diameter, assembled by a family of the three pore-forming proteins of the immune system. The three pore formers are complement component C9 secreted into blood and interstitial fluid, Perforin-1 expressed in cytotoxic T cells and natural killer (NK) cells, and Perforin-2 expressed by all phagocytic and all non-phagocytic cells examined to date. C9, Perforin-1, and Perforin-2 share the conserved membrane attack complex perforin (MACPF) domain, which functions in these proteins as a pore-forming killer domain (Figure 1) (1–3). These MACPF domains consist of 316–372 amino acids and although they share low overall homology (<50%) they contain the signature motifs that define them as members of the MACPF family (see Prosite entries PS00279 and PDOC00251 at http://prosite.expasy.org).

Pore formation by the MACPF ensues through homopolymerization of several monomers, which causes their MACPF domains to refold during polymerization to a hollow cylinder of 16 nm in length and an internal diameter of 10 nm for C9, 16 nm for Perforin-1, and 10 nm for Perforin-2 (4–14). Refolding of the MACPF domain during polymerization exposes hydrophobic sites that insert 5 nm deep into the hydrophobic interior of membranes or bacterial cell walls (4). Polymerization of the pore former is self-limiting by closure of the cylindrical complex that is anchored by its 5 nm long hydrophobic segment in the hydrophobic interior of the cell membrane or bacterial envelope, respectively. The inside of the cylinder is hydrophilic, creating a 10–16 nm wide, water-filled pore, reaching 5-nm deep into the bacterial membrane core or breaching the cell membrane. It is important to note that during immune attack large clusters of pores are formed on the target membrane (analogy machine gun) by all the three pore formers. The dense clusters disrupt large surface areas of the bacterial envelopes rendering them susceptible to secondary attack by ancillary agents, such as reactive oxygen and nitrogen species, lysozyme, and proteases. In the absence of Perforin-2, the ancillary effectors cannot kill pathogenic bacteria (14). Similarly, Perforin-1 pores are required for the penetration of granzymes to help kill virus-infected and cancer cells.

### The Discovery of the Pore-Forming Protein of Complement: Poly-C9

While studying purified human C9, Eckhard unexpectedly observed that C9 lost its hemolytic activity – a measure of complement function – at concentrations greater than 1 mg/ml. He subsequently employed electron microscopy and analytical ultracentrifugation, among other techniques, to determine the reason for this unexpected property (4, 15). With the electron microscope, he observed structures (1) that were nearly identical to the images of the entire C5b–9 membrane attack complex (MAC) that had been previously reported (4, 16). Yet, Eckhard’s samples were *comprised solely of C9*. Thus, Eckhard correctly deduced that the cylinder of the MAC C5b–9 must be composed primarily of polymerized C9.

Eckhard’s report, coauthored with Jurg Tschopp in 1981, of pore formation *via* polymerization and refolding of a single protein species (C9) was likely the first demonstration and molecular understanding of the molecular mechanism of pore formation (17). He had also found the answers to the two questions that had initially intrigued him when he joined Müller-Eberhard’s laboratory: what is the molecular mechanism by which complement kills invasive bacteria? and which one of the complement components is the killer molecule? For the former, he had shown that it was polymerization of C9, and for the latter, he identified C9 as the pore-forming killer molecule of complement (15, 17, 18).

At high concentrations, purified C9 in solution spontaneously polymerizes at 37°C or room temperature or very slowly at 4°C (4, 15). Polymerized C9 (poly-C9) sediments at 27S in the analytical ultracentrifuge, monomeric C9 at 4.5S. On SDS page, the apparent molecular weight increases from 70,000 Da for the C9 monomer to ~1,000,000 Da for poly-C9. Electron-microscopic images reveal that poly-C9 assumes the shape of a 100 Å-wide, 160 Å-long hollow cylinder that has a 5-nm long hydrophobic domain on one end that transverses lipid bilayers. Monomeric C9 is a globular, elliptic protein with axes length of ~5 and 8 nm (4). The cylindrical structure of poly-C9 closely resembles the structure of the entire C5b–8–poly-C9 complex, the MAC. The C5b–8 subunits are arranged into a narrow rod shaped, heteromeric complex that contributes little to the overall structure of the MAC-cylinder, even though it is integrated into the poly-C9 complex (18). The important function of C5b–8 is to trigger C9 polymerization and direct its membrane attack to the proper target, the bacterial surface. Although C5b–8 generates small transmembrane channels in cells and lyses red blood cells, it is insufficient to kill and lyse bacteria that have a thick outer cell wall. For killing of bacteria, C9 polymerization is required, which allows lysozyme access to the proteoglycan layer and causes the collapse of bacteria, most likely through the digestion of proteoglycan (19). To avert collateral damage by complement, our own cells are protected by CD55, preventing C3 binding, and CD59 preventing C8 binding and C9 polymerization. In addition, blood contains the S-protein also known as vitronectin that binds the MAC in solution and prevents C9-polymerization to prevent bystander lysis of our cells (5, 6, 20). Acquired deficiency of the complement protective proteins on our cells causes paroxysmal nocturnal hemoglobinuria (PNH), which is lethal if untreated (7–9).

### Insights and Inspirations from the Discovery of Poly-C9

The bilayer structure of cell membranes was discovered after electron microscopy had been developed in the 1950s. In 1964, Borsos et al. described electron microscopic “complement lesions” of about 100 Å diameter on the membranes of erythrocytes lysed by complement (10). Similar complement lesions were subsequently found also on cell walls of bacteria killed by complement (11). In 1972, Mayer proposed in a theoretical paper that membrane lesions represent rigid structures formed by C5, C6, C7, C8, and C9 of complement that look like donuts with a central hydrophilic hole (21). He also considered a donut endowed with enzymatic activity generating a leaky patch by enzymatically degrading membrane lipids or other components. The donut is a symmetrical ring, and it remained unexplained how the five different protein molecules, such as C5, C6, C7, C8, and C9, may be arranged to form such a symmetrical structure. Tranum-Jensen et al. in 1978 isolated the C5b–9 complex from erythrocyte membranes and showed that (*direct quote*), “The classical complement ‘rings’ visualized on membranes after complement lysis represent such C5b–9 cylinders perpendicularly oriented on the membranes” (16). The images confirmed the prediction of Mayer in which the donut is composed of C5–9 and extended the analysis to show that the donut is a hollow cylinder with an annulus forming the donut on one end.

The identification of C5b, C6, C7, C8, and C9 within the cylindrical complex remained a vexing riddle, given the symmetry of a cylinder and the diversity of the proteins forming it. This question was finally resolved with the discovery that isolated and purified C9 spontaneously polymerizes *to poly-C9*, forming a cylindrical complex that has an almost identical appearance as the C5b–9 MAC (4). C5b–8 forms a long rod-like structure attached to the bacterial membrane that triggers C9 to polymerize and form the C5b–8–poly-C9 cylinder (MAC) that is detected as complement lesion in the membrane. C5b–8 is integrated as a subunit into the polyC9 cylinder (22).

### Pore Formation Is Driven by Physico-Chemical Forces Not Requiring Enzymatic Activity

The fact that isolated, purified C9 spontaneously polymerizes in solution to a cylindrical complex suggests that the driving force for refolding of C9 and exposure of its hydrophobic domain even in aqueous solution is driven by physical interaction of C9 monomers during polymerization. The chain reaction of polymerization is self-limiting through the formation of a hollow cylinder. Intermediate polymers (half rings, representing a half cylinder sectioned along its long axis) are detected by electron microscopy when C9 is limited or is obstructed by other membrane components.

**Figure 2:**
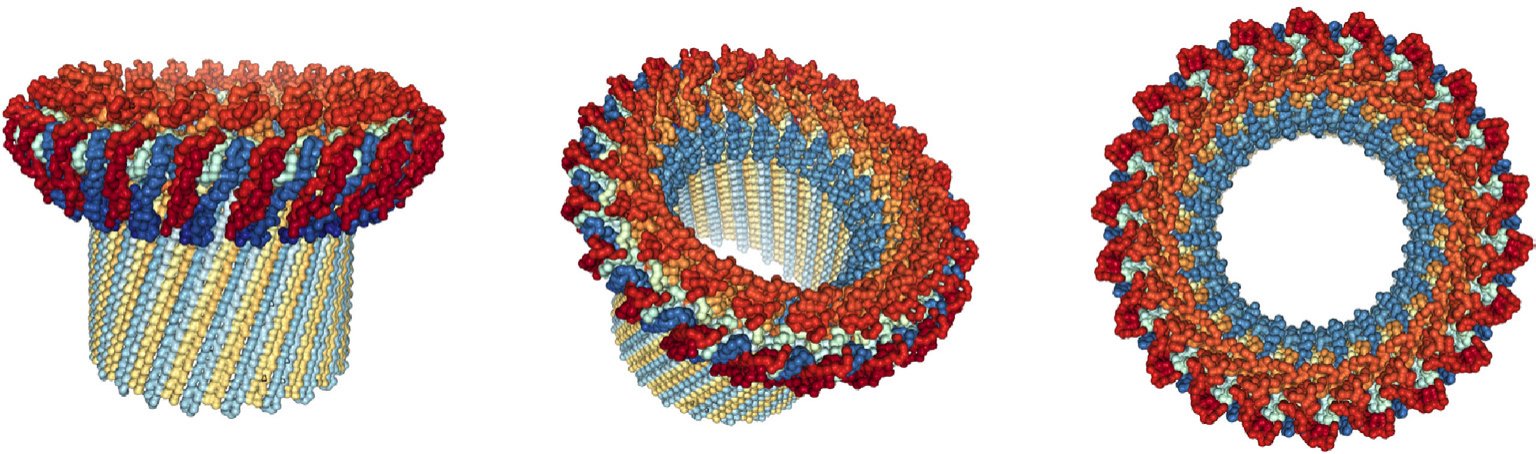
**Structure of poly-C9 at 8 Å resolution**. Side, tilted, and top views of soluble poly-C9 obtained by cryo-EM. In this reconstruction, 22 C9 molecules polymerize to form a symmetrical structure with globular domains atop a membrane penetrating 88 stranded β-barrel with a diameter of 120 Å. Graphic representations were derived from PDB file 5FMW and rendered with NGL Viewer (27).

The C9-polymer is likely formed by the stepwise addition of C9 monomers that unfold and insert into lipid. Each added unfolding C9 monomer is hydrophobic on the outside allowing lipid binding and is hydrophilic on the luminal side, repelling lipid from the inner side of the forming cylinder. The hydrophilic domains thereby repel lipids, which will be replaced by water and its solutes. In this way, we suggest, a water-filled, 100 Å wide pore is inserted solely by physical forces literally pushing hydrophobic lipids away. This analysis generated the first molecular model of pore formation by a single protein in 1982 (15). Eckhard’s model has recently been confirmed and refined in more detail by crystallization of the MACPF domain and detailed molecular studies (13, 23–28). Most recently, cryo-electron microscopy has revealed the structure of soluble poly-C9 at a resolution of 8 Å (Figure 2) (29, 30). This latter structure is composed of 22 C9 monomers forming a pore of 120 Å, close to Eckhard’s original measurements. The poly-C9 pores are used by additional ancillary antimicrobial agents to attack sensitive sites in the bacterial envelope. Only when C9 is added to the C5b–8 complex, can lysozyme cause the collapse of bacterium most likely by degradation of the proteoglycan layer that forms the “skeleton” of the bacterium (19).

It may not be too farfetched to compare the firing of poly-C9 by C5b–8 with a bullet fired by a gun. Poly-C9 blows a large, by molecular standards, pore (hole) into membranes, just like bullets make holes into targets by physical force. Bullets are fired by pulling the trigger of the gun, after the gun has been aimed at the target. Likewise, triggering C9 polymerization is accomplished by C5b–8, but it is the bullet C9 that hits the target. C5b–8 is assembled on the target bacterium by activated C3 that assumes the function of recognition and aiming. C9 is blind to the target it hits. It would kill our own cells just as well as bacteria, if our cells were not protected by the CD55 and CD59 proteins to block C3b activation and C9 polymerization, respectively. Acquired deficiency of these protective molecules causes PNH due to uncontrolled complement lysis of our cells (collateral damage) that can be lethal if untreated (7–9).

### The Discovery of *Perforin-1* in Natural Killer Cells and Cytotoxic T Lymphocytes

Natural killer cells kill cancer cells. Cytotoxic T lymphocytes (CTL) kill virus-infected cells and cancer cells after activation, clonal expansion, and differentiation of naive CD8^+^ T cells into CTL. The molecular mechanism of how NK cells or CTL kill their target cells was not known before 1983, and the T cell receptor was first cloned in 1984 (31). Many hypotheses suggested that secreted factors – lymphotoxin, TNF (cachectin), and NK cytolytic factor (NKCF) – were mediating cytotoxic activity (32–34). Another hypothesis invoked a role for ATP in killing (35). Yet, another hypothesis considered that the firm attachment between CTL and target caused target cell membrane shearing resulted in death of the target (36, 37). Finally, a popular hypothesis suggested that CTL can express complement proteins C5–C9 for use in killing of target cells.

Eckhard considered the hypothesis that killer lymphocytes may be endowed with a pore-forming killer protein similar to C9 of complement, making pores that can be detected by negative staining electron microscopy at 50,000-fold or higher magnification. In 1982, Eckhard initiated a collaboration with his friend Dr. Gunther Dennert at the Salk institute in La Jolla – who at the time was culturing and cloning NK cells – to determine whether or not NK cells use a pore-forming protein to kill their target cells. They subsequently found that mixing NK cells with tumor cells as targets and adding concanavalin A-induced killing of the tumor cells. Isolating membranes from the mixture and examining them by negative staining in the electron microscope revealed clusters of membrane pores similar to – but not identical to – poly-C9 of complement. NK cell-mediated pores have a diameter of 16 nm and project 12 nm above the membrane. The ultrastructure suggested a polymeric composition of about 14–16 protomers. Assuming 5-nm deep membrane insertion, the cylindrical or tubular complex thus had the dimensions of 16 nm length with an internal diameter of 16 nm. One end of the cylinder bears a 4- to 5-nm long hydrophobic domain which is membrane inserted. The other end of the cylinder bears an annulus that resembles a donut when viewed from the top.

Based on the morphology of the polymers ultrastructure and lymphocytic expression, it was clear to Eckhard and Gunther that they had identified a new, previously unknown protein. In reference to its membrane perforating ability, they named the monomeric protein Perforin-1 and its cylindrical polymer, poly-Perforin-1. They submitted their findings to *Nature* and the reviewers suggested publication. The Senior Editor of Nature agreed with the condition that they remove the name Perforin-1 and poly-Perforin-1. The editor insisted that the names were premature and unwarranted. Although Eckhard and Gunther did not necessarily agree with the editor, they reluctantly replaced Perforin-1 and poly-Perforin-1 with the descriptive terms “tubule” and “tubular structure” so as not to delay publication (12). Henkart and Dourmashkin had previously shown lesions on target cells killed by antibody-dependent cytotoxicity that were larger than those of complement (38). It is likely that these lesions were made by Peforin-1, called cytolysin by Henkart’s group, but the nature of the killer cell remained unclear. Thus, Eckhard and Gunther’s Nature paper was the first to define the molecular killing mechanism used by a bona fide cloned lymphocyte (NK cell) (12). Today, the moniker “Perforin-1” is now officially and broadly accepted. However, its initial rejection by the editor of Nature has caused the foundational paper on the subject to be regularly overlooked, because it is not revealed by the search term “perforin.” Eckhard felt that this invisibility had adverse consequences for his career and his research team during those pivotal years. This experience shaped Eckhard’s discussions with reviewers and editors who initially challenged his use of “Perforin-2” – the third pore-forming protein discovered over the course of his career – rather than the official name of MPEG1.

The name Perforin-1 was accepted by the editor in Gunther and Eckhard’s second paper on the subject in the Journal of Experimental Medicine (39). This paper revealed that CTL used the exact same pore-forming mechanism for the killing of target cells as did NK cells. For many T cell immunologists, the claim that the sophisticated T cell should use the same primitive mechanism, pore formation, for killing as do NK cells and complement seemed unlikely and was not accepted for the next decade (40).

### Perforin-1 Is Contained in Cytolytic Granules

Both Henkart’s and Eckhard’s groups set out to further define the molecular mechanism by which Perforin-1 is activated and kills target cells (41, 42). Henkart’s team used a rat large granular lymphocyte tumor cell line to isolate cytoplasmic granules. Eckhard’s team had noticed and commented on the proximity in electron micrographs of cytoplasmic granules of killer lymphocytes (NK and CTL) to the contact site (synapse) with the target cell (12). The images suggested the hypothesis that the granules may contain the killer protein Perforin-1. Eckhard’s group used the murine CTL line CTLL2 to purify cytoplasmic granules. This cell line had been established by Kendall Smith and was widely used to titrate interleukin-2 (IL-2) (43). At the time (1983), the cells required only IL-2 for growth without the need for TCR stimulation. Electron micrographs revealed that CTLL2 contained many large cytoplasmic granules. CTLL2 cells were therefore chosen to isolate and analyze granules and their potential cytolytic activity (44).

Granules from CTLL2 in the presence of calcium ions are highly lytic for all tumor cell lines tested. Lysis is complete within less than 30 min. Granules also lyse red blood cells within 5 min allowing for a rapid assay by hemolysis. Lysis is strictly Ca^2+^ and temperature dependent. There is no lysis in the absence of calcium ions or at 4°C. The finding that granules from CTLL2 could lyse all kinds of tumor targets and red cells, suggested that they are unspecific in regard to the type of target they lyse. The specificity of CTL-mediated target lysis therefore must come from the CTL containing the granules that are released after contact with the target *via* the T-cell receptor.

Thus, in analogy to the pore-forming protein C9, the bullets fired by the CTL or NK cell, Perforin-1, are blind as to the nature of their target. The targeting is accomplished by the CTL receptor or by NK receptors. Upon contact with the target cell, the granules of the killer cells are moved *via* microtubules to the contact site and released (exocytosed) onto the target membrane. Exocytosis into the interstitial space brings the granules into a Ca^2+^-containing environment which induces Perforin-1 polymerization and insertion of clusters of poly-Perforin-1 pores in target cell membranes. The pores are used by granzymes A-M that are contained in the same granule, to enter the cell and help killing by enzymatic action on several substrates that lead to DNA degradation and cell death. A low number of pores allow cells to repair the membrane, but entry of granzymes through the pore prevents survival. Large clusters of poly-Perforin-1 pores kill the target cell without ancillary granzymes.

### Cloning of Perforin-1 and Comparison to C9, Identification of the Membrane Attack Complex Perforin Domain

In the early 1980s, molecular biology was a new field. Cloning and sequencing projects could easily take a year or more. Eckhard began a collaboration with the Zinkernagel/Hengartner laboratory in Zurich, Switzerland, to clone Perforrin-1. This group was cloning the T-cell receptor specific for the lymphocytic chorio-meningitis virus (LCMV) using a lamda-gt-11 protein expression library. This bacteriophage was used to introduce cDNA into *Escherichia coli* and drive protein expression of the cloned DNA. This allowed screening of lysed bacterial colonies with antibodies for the protein of interest. Using an anti-Perforin-1 antiserum prepared in house (monoclonal antibodies were not yet available), the collaborators screened the phage library for Perforin-1. This strategy eventually led to the successful cloning and sequencing of both human and mouse Perforin-1 (45, 46). At the same time, a group from Japan published the sequence of mouse Perforin-1 by using oligonucleotide screening based on partial N-terminal Edman degradation of the protein and reverse translation (47). Eckhard’s team had previously also cloned and sequenced C9 in collaboration with Fey and DiScipio (42, 48). Both the Japanese and Eckhard’s group noted a sequence (domain) that was similar between C9 and Perforin-1. This common domain was subsequently officially named the MACPF domain. The discovery that C9, a single protein, can form membrane pores created a new paradigm of pore formation. Today, over 500 prokaryotic and eukaryotic proteins with MACPF domains are known and may form pores by identical principles – a protein is triggered to polymerize, refold, insert, and create pores in membranes. The targets of the MACPF domain are very diverse as are the triggers for polymerization.

### The Biological Function of Perforin-1

Despite the published sequence of Perforin-1, there remained considerable doubt among T-cell scientists about the biological importance of Perforin-1 *in vivo*. To address these doubts, Eckhard decided to knockout the gene for Perforin-1 in mice by homologous recombination, a technique first reported (49). Eckhard collaborated with Burki (Sandoz Pharma) and the Zinkernagel/Hengartner laboratory (Zurich) to publish the Perforin-1 knockout mouse in 1994 (50). Perforin-1 knockout mice are viable and fertile and have normal numbers of CD8^+^ T cells and NK cells. However, these cells do not lyse virus-infected cells, allogeneic fibroblasts, nor NK target cells *in vitro*. The Perforin-1 knockout mice also fail to clear LCMV *in vivo*, and they eliminate tumor cells with reduced efficiency (51). Perforin-1 is therefore a key effector molecule for T-cell- and NK cell-mediated cytolysis of virus-infected and cancer cells. For Eckhard, the observation that Perforin-1 in CD8^+^ CTL and NK cells may be involved in killing tumor cells was also personally important. As a physician, he had a particular interest harnessing the power of the immune system to defeat cancer and Perforin-1 in NK cells and CD8^+^ CTL seemed to offer a path forward (52).

The Nature publication finally convinced the scientific community of the biological importance of Perforin-1, the second pore-forming protein of the mammalian immune system to be described (50). In 1999, the first human Perforin-1 deficiency was reported (53). The deficiency caused familial hemophagocytic lymphohistiocytosis (FHL), a rare and rapidly fatal autosomal recessive immune disorder characterized by uncontrolled activation of T cells and macrophages, as well as overproduction of inflammatory cytokines. This latter report validated the biological importance of Perforin-1 in humans.

### The Discovery of Perforin-2

The common MACPF domain of C9 and Perforin-1 inspired Eckhard to Blast-search the NCBI database frequently with the consensus sequence of the MACPF domains of both pore-forming proteins. Possibly, additional MACPF-containing proteins could be found that are not complement proteins or Perforin-1. Success came when expressed sequence tags (ESTs), a method pioneered by Craig Venter at the NIH, were made publicly available by the NCBI in 1995. Within the ESTs, Eckhard found several that contained a novel MACPF domain. Following the source of the MACPF ESTs, it seemed that the new MACPF protein is expressed in macrophages. Eckhard assembled the complete open reading frame of the novel protein from several overlapping ESTs. The predicted protein appeared to be a 78-kDa protein containing a signal peptide followed by a MACPF domain at its amino terminus (Figure 1). It also contains a predicted transmembrane domain near its carboxy terminus. The predicted protein therefore was a type 1 membrane protein with a short cytosolic domain. This latter feature distinguished the new MACPF protein from the secreted polypeptides Perforin-1 and C9 (Figure 1).

Concurrent with Eckhard’s discovery of a new MACPF encoding gene in ESTs a manuscript was published in *Blood* describing a gene that is expressed during the differentiation of monocytes to macrophages. This novel gene was named macrophage-expressed gene 1 (*Mpeg1*) (54). The *Mpeg1* encoded protein described by Spilsbury et al. was largely identical to Eckhard’s MACPF protein, with the exception that Spilsbury’s predicted protein (GenBank accession AAA73957) lacked the transmembrane and cytosolic domains of Eckhard’s. This omission is most likely the result of a sequencing error as the domains are present in other GenBank records, for example, accession AAI12231 (human), XP_009184227 (baboon), XP_508450 (chimpanzee), Q2M385 (mouse), and NP_001292389 (rat). Because the MACPF domain precedes the apparent sequencing error, Spilsbury et al. were able to note that the product of the *Mpeg1* gene shares distant ancestry to Perforin-1, the lytic protein found in CTL and NK cells (54). Surprisingly, to the best of our knowledge, the authors did not publish follow-up studies with their novel gene.

### Perforin-2 and Pore Formation

Given the absence of functional analyses of this novel gene, Eckhard’s first objective was to determine if it was a pore-forming protein. To accomplish this, Eckhard’s group transfected various cell lines with plasmids expressing MPEG1–GFP fusion proteins and transfected various cell lines with the construct looking for GFP fluorescence. However, GFP^+^ cells would invariably die suggesting that the fusion protein was cytotoxic. Finally, after a year of experimenting, they found that HEK-293 cells were able to tolerate the fusion protein and survive. With the necessary cell line and reagents in place, the group was able to use electron microscopy to image pore-like structures in the membranes of transfected cells and in the membranes of bacteria exposed to cells expressing MPEG1 (14). Moreover, Eckhard and collaborators found ample evidence that the pore-forming protein was a killer, bactericidal protein (1, 14, 55, 56). These observations supported Eckhard’s hypothesis that MPEG1 kills its targets by forming clusters of pores on target membranes, similar to C9 and Perforin-1. The analogy to Perforin-1 also led Eckhard to advocate for the renaming MPEG1 to Perforin-2 because he felt that the latter moniker was more descriptive of its function than the former.

Macrophages phagocytose bacteria, especially when opsonized by complement, and kill bacteria intracellularly. The killing mechanism had been well established by 1995. According to the dogma at the time – also taught in textbooks – phagocytosed bacteria are killed by reactive oxygen and nitrogen species, by acidification, and lysosomal enzymes after fusion with the lysosome. According to this model, the killing of intracellular bacteria in macrophages does not require a pore-forming protein such as Perforin-2. This led Eckhard and his collaborators at the University of Miami to ask the following question – If macrophages already have perfectly good killing mechanisms why do they express Perforin-2? The answer was surprising and apparently paradigm shifting. The researchers found that macrophages are unable to kill phagocytosed bacteria (methicillin resistant *Staphylococcus aureus, Salmonella typhimurium, Enteropathogenic E. coli, Mycobacterium smegmatis, Mycobacterium tuberculosis, Yersinia pseudotuberculosis, Chlamydia trachomatis*, and other species) when Perforin-2 is knocked down by siRNA. Instead, the bacteria survive and replicate intracellularly (1, 14, 56). Moreover, killing was restored by transfection of Perforin-2 expression plasmids lacking the siRNA targeting regions (1, 14).

Further studies revealed that interferons induce the expression of Perforin-2 in fibroblasts, thus endowing them with the ability to kill invasive, intracellular pathogens (56). Interferon inducible or constitutive expression of Perforin-2 has been observed in all cell types analyzed to date (*n* ≥ 70) (14). This raises the possibility that Perforin-2 is used by every cell in our bodies to kill pathogenic bacteria. Moreover, killing is not limited to intracellular compartments as we have shown that Perforin-2 can also kill extracellular bacteria attached to the cytosolic membrane (1). Eckhard also hypothesized that Perforin-2 may also protect against enveloped and/or endocytosed viruses. This hypothesis is currently under investigation by Eckhard’s collaborators at the University of Miami. Regardless of the outcome of this latter hypothesis, several studies by Eckhard and his collaborators have demonstrated that Perforin-2 is pivotal for the control and destruction of both intra- and extracellular bacteria (1, 14, 55–57).

### The Essential Role of Perforin-2 *In Vivo*

With several *in vitro* studies confirming Eckhard’s hypothesis that Perforin-2 was a pore-forming bactericidal protein, he invested resources into the generation of Perforin-2 knockout mice. Perforin-2^−/−^ mice are born healthy and do not require gnotobiotic conditions. Rather, they are colonized by commensal microorganisms without ill effects and develop normally under pathogen-free conditions. Thus, Perforin-2 is neither required for development nor for the control of commensals. However, Eckhard and his colleagues found that Perforin-2 knockout mice are highly susceptible to pathogenic bacteria even at low infectious doses. For example, Perforin-2^−/−^ mice suffer progressive weight loss and perish 15–20 days after orogastric inoculation of just 10^5^ CFU of *Salmonella enterica* serovar *typhimurium* (14). In contrast, wild-type mice suffered only transient weight loss and the majority of animals survived. Perforin-2 heterozygotes displayed a gene dosage effect with more severe weight loss and lower survival rates than wild-type littermates but not as severe as Perforin-2 knockout mice (14). In addition, Perforin-2 deficiency correlated with greater pathogen dissemination and higher bacterial loads in the blood, spleen, liver, and other organs. Similar patterns were observed after orogastric inoculation of *Y. pseudotuberculosis* or epicutaneous challenge with MRSA (1, 14). Thus, several *in vivo* and *in vitro* studies by Eckhard and his collaborators have now shown that Perforin-2 is a pore-forming protein and potent bactericidal molecule charged with limiting the proliferation and spread of infectious microbes (1, 14, 55–57).

### Activation of Perforin-2 and Anti-Perforin-2 Effectors

Perforin-2 is found in vesicles that stain with markers for plasma membrane, early endosomes, endoplasmic reticulum, Golgi, and the post-Golgi network (14). As a type I transmembrane protein, the orientation of Perforin-2 would place its MACPF domain in the lumen of intracellular compartments or the extracellular space if in the plasma membrane. In the absence of a pathogen-associated molecular pattern (PAMP), the overall cellular distribution of Perforin-2 is diffuse and perinuclear as determined by confocal microscopy of host cells transfected with Perforin-2–RFP expression plasmids (1). However, LPS – and presumably other PAMPs – or live bacteria trigger the ubiquitylation of Perforin-2’s cytosolic tail by a cullin-RING E3 ubiquitin ligase (CRL) followed by a rapid redistribution of Perforin-2 into distinct punctate bodies (1). Mutagenesis of conserved lysine residues in the cytosolic tail of Perforin-2 abolishes its redistribution and Perforin-2-dependent killing (1). Thus, our observations suggest a model wherein ubiquitylation triggers the translocation of Perforin-2 laden vesicles to phagosomes and/or the plasma membrane. Subsequent membrane fusion delivers the MACPF domain to the lumen of phagosomes or extracellular space whereupon it polymerizes to perforate the envelope of phagocytosed bacteria or extracellular bacteria at the plasma membrane.

The importance of Perforin-2 to host defense is further supported by its conservation throughout evolution and its broad distribution across the animal kingdom from invertebrates to vertebrates (2, 3, 58, 59). However, if Perforin-2 is a potent and effective pore-forming protein, why do infectious diseases exist? The long evolutionary history of Perforin-2 suggests that pathogens have had ample time to evolve mechanisms to suppress or evade its bactericidal activity. We have recently validated this prediction by showing that enteropathogenic *E. coli* and *Y*. *pseudotuberculosis* inhibit Perforin-2-dependent killing by blocking its intracellular trafficking (1). This is accomplished by the bacterial effector protein Cif which is injected into the cytosol of host cells by the bacteria whereupon it proceeds to deamidate NEDD8 (60–62). NEDD8 is a member of the ubiquitin family of proteins and neddylation is essential for CRL-dependent ubiquitylation of CRL substrate proteins (63, 64). Thus, Cif blocks Perforin-2-dependent killing by inhibiting its ubiquitylation through deamidation of NEDD8 (1). Although only a few species of pathogenic bacteria express Cif, we suspect that evolution may have endowed most bacterial pathogens with the ability to inhibit or resist the bactericidal activity of Perforin-2. The identification of drugs that restore and/or increase Perforin-2 activity may overcome these anti-Perforin-2 effectors and allow our own bodies to successfully combat the invaders. These new approaches may also provide a solution to the threat of widespread antibiotic resistance.

## Summary and Conclusion

Over the course of his scientific career, Eckhard Podack discovered and characterized three pore-forming proteins that are essential to protect our lives from bacteria, viruses, and cancer.

Complement and poly-C9 kill extracellular pathogens in blood and interstitial fluid. Although C9 deficiency is not lethal, it is associated with increased risk of chronic, recurring infection with *Neisseria meningitides* and *Neisseria gonorrhea* (65, 66). This suggests that *Neisseria* have mechanisms to block Perforin-2, but not poly-C9, which can kill them. Most other extracellular pathogens are detected by complement by one of its three activation pathways as well as additional proteins such as C-reactive protein (CRP). Complement activation-specific antibody and C3b deposition leads to opsonization and avid phagocytosis with subsequent intracellular killing, presumably by Perforin-2.

Peforin-1 kills virus-infected cells and cancer cells. Human Perforin-1 deficiency causes familial hemophagocytic lymphohistiocytosis, which is lethal if not treated. It also protects us from cancer. All solid tumors have a mechanism to suppress CD8^+^ CTL production or action, thereby neutralizing Perforin-1 activity. It is possible that methods overcoming cancer-induced neutralization of Perforin-1 through suppression of CD8^+^ CTL induction or activity, in combination with tumor-specific cancer vaccines that generate CD8^+^ CTL, will allow remission or even cure of cancer.

Perforin-2 kills both intra- and extracellular bacteria. Unlike poly-C9 which is restricted to pore formation in the outer membrane of Gram-negative bacteria, Perforin-2 is a broad-spectrum killer of Gram-positive, Gram-negative, and acid-fast pathogenic bacteria, including those that are antibiotic resistant. Its transmembrane domain further differentiates Perforin-2 from the other two pore-forming proteins of the immune system; however, it remains to be determined if cleavage from the membrane precedes polymerization and pore formation. Based on our observations with Perforin-2 knockout mice which are hypersusceptible to even low doses of bacterial pathogens, it is likely that individuals with complete Perforin-2 deficiency succumb to infectious disease early in childhood. This would have been especially true prior to the widespread availability of antibiotics. Individuals with Perforin-2 haplo-insufficiency may reach maturity but are likely to suffer higher rates of chronic and recurring infectious disease than the general population. Eckhard initiated a collaboration with Dr. Holland (NIH) to test this latter hypothesis and the analysis is ongoing.

The three pore-forming proteins of the human immune system therefore are essentially life-saving proteins protecting us from microbial invasion and providing surveillance against cancer formation.

## Author Contributions

EP (1943-2015) wrote the original version of this manuscript shortly before his death. Kendall A. Smith edited and formatted the manuscript that was submitted posthumously for EP. The reviewed MS was subsequently edited and revised by GM to address reviewer comments and to include additional data regarding Perforin-2.

## Conflict of Interest Statement

The authors declare that the research was conducted in the absence of any commercial or financial relationships that could be construed as a potential conflict of interest.
